# Associations of Problematic Binge-Watching with Depression, Social Interaction Anxiety, and Loneliness

**DOI:** 10.3390/ijerph18031168

**Published:** 2021-01-28

**Authors:** Jia-Ji Sun, Yen-Jung Chang

**Affiliations:** Department of Health Promotion and Health Education, National Taiwan Normal University, No. 162, Section 1, Heping E. Road, Taipei 106, Taiwan; jaimao2812@gmail.com

**Keywords:** problematic binge-watching, depression, social interaction anxiety, loneliness

## Abstract

Background: Binge-watching refers to the watching of several episodes of a TV series or program in rapid succession. This study aims to investigate the associations of binge-watching behavior with depression, social interaction anxiety, and loneliness risks among adults in Taiwan. Methods: A cross-sectional online survey was conducted in October 2018, in which data from 1488 participants were collected using a self-administered questionnaire comprising four valid and reliable scales: the Center for Epidemiologic Studied Depression Scale (CES-D), the Chinese version of the Social Interaction Anxiety Scale (SIAS-C), the UCLA Loneliness Scale (version 3), and the Problematic Series Watching Scale (PSWS). Results: Among the surveyed participants, the mean age was 28.3, and most participants were women who had completed undergraduate education. Multiple regression analysis demonstrated that, after adjustments for sociodemographic characteristics and self-reported health statuses, the score on the problematic binge-watching scale was positively associated with the scores on the depression, social interaction anxiety, and loneliness scales (*p* < 0.001 for each model). Conclusions: Problematic binge-watching was associated with increased depression, social interaction anxiety, and loneliness risks among adults in Taiwan. Additional studies on the relationship between problematic binge-watching and mental health problems, as well as its potential mechanism, are warranted.

## 1. Introduction

### 1.1. Emergence of Binge-Watching Behavior

Recent advancements in technology have led to the emergence of a new phenomenon called binge-watching, which refers to the watching of several episodes of a TV series or program in rapid succession. According to a recent survey in the United States, approximately 70% of the surveyed TV viewers engaged in binge-watching, and they watched an average of five episodes per binge-watching session [1]. Binge-watching is an emergent problem, and its health implications have not been explicitly studied thus far. It has been proposed that the symptoms of binge-watching behavior meet the diagnostic criteria for addiction; hence, it could be considered an addictive behavior [2]. On the basis of the notion of behavioral addiction, the mental health issues related to binge-watching could be a major concern.

### 1.2. Binge-Watching and Mental Health

In recent years, the relationship between binge-watching and mental health has been under scrutiny, but the findings are debatable. Individuals with binge-watching behavior are more likely to be affected by depression because depression makes people want to escape their current state of frustration and consume more TV to release this pressure [3]. Individuals with depressive symptoms and low self-regulation tend to engage in binge-watching to distract themselves from their negative emotions [4]. By contrast, other studies have proposed that binge-watchers felt more relaxed and happier after watching TV shows, indicating that the binge-watching frequency is negatively correlated with the depression level [5,6].

The association between binge-watching and anxiety has also been studied. Individuals with anxiety tend to relax and pass time by binge-watching [7], and adults with anxiety symptoms are likely to spend more time watching TV than the general population [8]. However, anxiety linked to social interaction has received little research attention. Social interaction anxiety refers to the distress experienced when interacting with other people in a social environment, which stems from worry or nervousness about what to say or how to respond in social interactions [9]. Individuals who spend an excessive amount of time watching TV tend to become socially isolated, and that likely limits the development of their social networks and social skills [10]. Therefore, examining the possible association between binge-watching and social interaction anxiety is critical.

Loneliness is an inherent, subjective, negative emotional response resulting from negative external feelings triggered by the environment and other factors, such as personality traits and a lack of social connection [11]. The association between binge-watching and loneliness is debatable in the existing literature. A previous study highlighted that loneliness is positively related to binge-watching because viewers tend to watch more episodes to cope with loneliness [12]. Conversely, another study discovered no correlation between binge-watching and loneliness [3].

The binge-watching–mental health association remains unclear. In addition, the measurement of binge-watching presents another research gap. To assess binge-watching, most studies adopted various combinations of these indicators: (1) the frequency of binge-watching; (2) the duration of one viewing session; and (3) the number of episodes watched [13]. To date, there are only a few studies which proposed psychometrically validated instruments. The present study employed the Problematic Series Watching Scale (PSWS) [14] based on Griffiths’ behavioral addiction model [15] to examine the associations of problematic binge-watching with depression, social interaction anxiety, and loneliness risks.

## 2. Materials and Methods

### 2.1. Study Participants and Data Collection

A cross-sectional survey was conducted through an online survey system. Data were collected from 1488 participants aged ≥20 years in October 2018 by employing an anonymous and self-administered questionnaire. Purposive sampling was used. The institutional review board of National Taiwan Normal University approved the study procedure and research materials. Informed consent was obtained from all participants. No incentive was provided to participants.

### 2.2. Measurements

The questionnaire included five sections. The first section collected information on the study participant’s sociodemographics and self-reported health status. The other four sections assessed problematic binge-watching, depression, social interaction anxiety, and loneliness, respectively.

#### 2.2.1. Problematic Binge-Watching Behavior

The PSWS [14] was utilized to measure problematic binge-watching behavior using five point Likert items (Appendix A). This scale is based on Griffiths’ six-component addiction model, comprising salience, mood modification, tolerance, withdrawal symptoms, conflict, and relapse [15]. The scores ranged from 6 to 30, with a higher score indicating a higher problematic binge-watching risk. The Chinese version of the PSWS was translated and reviewed by expert meetings. In addition, this version of the PSWS was pilot tested to modify the wording to fit in with the sociocultural context of Taiwan. The Cronbach’s α for the PSWS was 0.83 in this study.

#### 2.2.2. Depression

Depression was assessed using the Center for Epidemiologic Studied Depression Scale (CES-D) [16], which was translated into Chinese [17]. The CES-D is a 20 item, 4 point Likert scale that assesses the presence of depressive symptoms. Scores were obtained by measuring the frequency of the symptoms per week (range: 0–60), with a higher score denoting a higher level of depression. The Cronbach’s α for the CES-D was 0.89 in this study.

#### 2.2.3. Social Interaction Anxiety

Social interaction anxiety was evaluated using the Chinese version of the Social Interaction Anxiety Scale (SIAS-C) [18], based on a 5 point Likert scale ranging from 0 to 80. A high score indicates an increase in anxiety levels. The Cronbach’s α was 0.90 in this study.

#### 2.2.4. Loneliness

Loneliness was examined using the UCLA Loneliness Scale (version 3) [19]. This scale is based on a 4 point Likert scale ranging from 20 to 80, with a higher score indicating a stronger perception of loneliness. The Cronbach’s α was 0.90 in this study.

### 2.3. Statistical Analysis

The data were analyzed using SAS (version 9.4; SAS Institute Inc., Cary, NC, USA). Multiple regression analysis was performed to investigate the association of binge-watching with depression, social interaction anxiety, and loneliness. Significance was set at *p* ≤ 0.05.

## 3. Results

### 3.1. Demographic Characteristics and Scale Scores

Among the 1488 surveyed participants, the mean ± standard deviation (SD) age of the participants was 28.3 ± 7.8 years. Most participants were women (74.9%) and had completed undergraduate education. More than half (55.4%) of the participants were employed full-time, and 58.3% were single. Approximately 29.2% and 45.6% of the participants reported good physical and mental health statuses, respectively. The mean (SD) scores on the UCLA Loneliness Scale, PSWS, CES-D, and SIAS-C were 39.3 (8.3), 15.3 (4.9), 14.9 (8.3), and 34.1 (14.6), respectively (Table 1).

Female participants had higher scores on the PSWS (*p* < 0.01 by the *t*-test), and those aged 40 or older had lower scores on it than their younger counterparts (*p* < 0.01 by the ANOVA test). Participants who reported poor physical or mental health had higher PSWS scores than those who reported a fair or good health status (*p* < 0.001 by the ANOVA test) (Table 2).

### 3.2. Problematic Binge-Watching and Mental Health

Table 3 shows the correlations between the PSWS score and the scores on the three mental health scales. The Pearson correlation coefficients indicated that participants engaging more in problematic binge-watching were more likely to report higher scores for depression, social interaction anxiety, and loneliness (r = 0.23, 0.22, and 0.22, respectively; all *p* < 0.001).

After adjustments for sociodemographic characteristics and self-reported health statuses, multiple regression analysis revealed that the problematic binge-watching score was significantly associated with the scores for depression, social interaction anxiety, and loneliness (β = 0.39, 0.55, and 0.35, respectively; all *p* < 0.001; Table 4, Table 5 and Table 6, respectively).

## 4. Conclusions

The findings of this study indicated positive associations between problematic binge-watching and depression, social interaction anxiety, and loneliness. The connection between binge-watching and mental health problems might be explained by considering binge-watching as an emotion-focused coping strategy [13]. Emotional enhancement and coping are key motives for binge-watching [20]. The current study’s findings support the positive association between binge-watching and depression reported previously [3,7,12]. It was suggested that binge-watching might serve as an easy way to escape reality and avoid negative emotions, which leads to a decrease in choosing other adaptive coping methods [21]. Individuals experiencing negative emotions and having difficulties applying adaptive coping strategies might tend to engage in excessive binge-watching as a coping approach [22]. Furthermore, individuals who depend on using media to regulate their restless moods might find it difficult to stop using media [23]. A lack of self-regulation can have a mediating effect on depression and media addiction, contributing to increased media consumption [24]. Furthermore, depression symptoms may also affect subjective time flow and result in the perception of time passing slowly [25]. This phenomenon might also lead to binge-watching.

Our findings also suggest that problematic binge-watching is associated with increased social interaction anxiety risk. Studies have indicated a positive association between screen time and anxiety [26,27]. However, to our knowledge, this study is the first to have investigated the association between problematic binge-watching and the risk of social interaction anxiety. Watching TV can serve as a means of relaxation for individuals affected by anxiety. Individuals with higher attachment anxiety may be more likely to watch an excessive amount of TV because of a sense of closeness to the characters on TV [7]. Moreover, watching TV may help viewers participate in virtual social interactions [28]. Hence, individuals feeling anxious regarding social interactions may seek virtual social interactions or relationships through binge-watching.

Contrary to several studies that have reported nonsignificant correlations between binge-watching and loneliness [3,4,29], this study reports that problematic binge-watching is associated with an increased risk of loneliness. This positive association may exist because viewers tend to watch an increasing number of episodes to cope with loneliness [12]. Furthermore, individuals who spend an excessive amount of time on screen may reduce face-to-face communication with family and friends, or likely experience social isolation and demonstrate limited development of social support networks [10,30], which also increases the risk of loneliness. Watching TV shows was previously believed to make viewers feel accompanied or entertained [31,32]. However, research on whether binge-watching connects to the feeling of loneliness is insufficient.

This is the first study to have examined problematic binge-watching behavior and mental health in the context of Taiwan. Valid and reliable scales were used to measure all primary research concepts: problematic binge-watching, depression, social interaction anxiety, and loneliness. However, this study has several limitations. First, the cross-sectional study design could not establish a causal inference of the association between binge-watching and mental health. Second, the study was a self-administered, questionnaire–based survey. Thus, it possibly suffered from recall bias. In addition, the study participants came from a convenience sample, and the study sample might not be representative of the entire adult population of Taiwan. Therefore, the results of the present study might be less applicable to the general population. Most of the surveyed participants in this study were women (74.9%). In most of the previous studies concerning binge-watching, it was observed that women comprised the majority of the research subjects [33].

Binge-watching remains a construct which lacks consensus regarding its definition and measurement [13,33]. Most previous studies have measured binge-watching by assessing self-reported TV watching time and frequency, while a few studies proposed psychometrically validated instruments [14,20]. This study employed the PSWS based on the Griffiths’ six-component addiction model to assess problematic binge-watching. The continuous accumulation of data using the validated instruments will improve the understanding of binge-watchers and the comparability of research results.

In summary, problematic binge-watching was found to be positively associated with depression, social interaction anxiety, and loneliness risks. Longitudinal research on the relationship between problematic binge-watching and mental health problems and its potential mechanisms is recommended.

## Figures and Tables

**Table 1:** Sociodemographics, Self-reported health statuses, and scores on the Problematic Series Watching and mental health scales.

	N	%	Mean	SD
Gender				
Male	374	25.1		
Female	1114	74.9		
Age			28.3	7.8
Education Level				
High School	35	2.4		
Bachelor’s Degree	1036	69.6		
Master’s Degree	417	28.0		
Employment Status				
Full-Time Employment	824	55.4		
Part-Time Employment	209	14.0		
Unemployed	455	30.6		
Relationship Status				
Married	212	14.3		
In a Relationship	408	27.4		
Single	868	58.3		
Self-Reported Good Physical Health	435	29.2		
Self-Reported Good Mental Health	678	45.6		
The PSWS Score (Range: 6–30)			15.3	4.9
The CES-D Score (Range: 0–60)			14.9	8.3
The SIAS-C Score (Range:0–80)			34.1	14.6
The UCLA Loneliness Scale Score (Range: 20–80)			39.3	8.3

**Table 2:** Scores on the Problematic Series Watching Scale (PSWS) by sociodemographics and self-reported health status.

	PSWS Score (Range: 6–30)		
	Mean	SD	d
Gender **			
Male	14.6	5.0	
Female	15.5	4.8	0.19
Age **			
20~29	15.3	4.9	
30~39	15.7	4.7	0.08
40 or older	14.1	4.9	−0.24
Education Level			
High School	14.8	5.0	
Bachelor’s Degree	15.5	4.9	0.14
Master’s Degree	14.9	4.8	0.02
Employment Status			
Full-Time Employment	15.3	4.8	
Part-Time Employment	14.8	4.9	−0.10
Unemployed	15.5	5.0	0.04
Relationship Status			
Married	15.1	5.0	
In a Relationship	14.9	4.7	−0.04
Single	15.5	4.9	0.08
Self-Reported Physical Health ***			
Poor	16.1	5.1	
Fair	15.2	4.6	−0.19
Good	14.4	4.7	−0.35
Self-Reported Mental Health ***			
Poor	16.9	5.2	
Fair	15.4	4.3	−0.32
Good	14.5	4.9	−0.48

** *p* < 0.01. *** *p* < 0.001. d = Cohen’s d.

**Table 3:** Pearson correlations between the PSWS score and scores on the mental health scales.

	PSWS Score	
	Pearson r	*p*-Value
The CES-D Score	0.23	<0.001
The SIAS-C Score	0.22	<0.001
The UCLA Loneliness Scale Score	0.22	<0.001

**Table 4:** Multiple regression model of depression, measured on the Center for Epidemiologic Depression Scale (CES-D).

	Β	SE	T
Female (Ref.: Male)	−0.83	0.53	−1.57
Age	−0.06	0.04	−1.57
Education Level (Ref.: Master’s Degree)			
High School	2.01	1.56	1.29
Bachelor’s Degree	0.52	0.52	1.00
Employment Status (Ref.: Unemployed)			
Part-Time Employment *	1.85	0.73	2.53
Full-Time Employment	−0.75	0.55	−1.36
Relationship Status (Ref.: Single)			
Married ***	−3.26	0.81	−4.00
In a Relationship **	−1.47	0.53	−2.77
Self-Reported Good Physical Health (Ref.: Bad/Fair)	−1.00	0.55	−1.83
Self-Reported Good Mental Health (Ref.: Bad/Fair) ***	−6.81	0.50	−13.57
The PSWS Score ***	0.39	0.05	8.26
F = 42.16; R^2^ = 0.23			

* *p* < 0.05. ** *p* < 0.01. *** *p* < 0.001. CES-D = Center for Epidemiologic Studied Depression Scale. Ref. = reference group. PSWS = Problematic Series Watching Scale.

**Table 5:** Multiple regression model of social interaction anxiety, measured on the Chinese version of the Social Interaction Anxiety Scale (SIAS-C).

	β	SE	T
Female (Ref.: Male)	−1.07	0.82	−1.30
Age ***	−0.36	0.06	−5.90
Education Level (Ref.: Master’s Degree)			
High School	2.22	2.41	0.92
Bachelor’s Degree	0.56	0.80	0.70
Employment Status (Ref.: Unemployed)			
Part-Time Employment	1.30	1.13	1.14
Full-Time Employment	−0.75	0.85	−0.88
Relationship Status (Ref.: Single)			
Married ***	−4.66	1.26	−3.69
In a Relationship ***	−3.27	0.82	−3.99
Self-Reported Good Physical Health (Ref.: Bad/Fair)	−0.84	0.85	−0.99
Self-Reported Good Mental Health (Ref.: Bad/Fair) ***	−3.69	0.78	−4.74
The PSWS Score ***	0.55	0.07	7.49
F = 24.98; R^2^ = 0.15			

*** *p* < 0.001. SIAS-C = Social Interaction Anxiety Scale, Chinese version. Ref. = reference group. PSWS = Problematic Series Watching Scale.

**Table 6:** Multiple regression model of loneliness, measured on the UCLA Loneliness Scale.

	β	SE	T
Female (Ref.: Male) ***	−2.22	0.58	−3.83
Age	0.05	0.04	1.19
Education Level (Ref.: Master’s Degree)			
High School	−0.22	1.71	−0.13
Bachelor’s Degree	0.96	0.57	1.69
Employment Status (Ref.: Unemployed)			
Part-Time Employment	1.20	0.80	1.50
Full-Time Employment	−0.84	0.60	−1.40
Relationship Status (Ref.: Single)			
Married ***	−4.51	0.89	−5.06
In a Relationship ***	−4.27	0.58	−7.37
Self-Reported Good Physical Health (Ref.: Bad/fair) **	−1.61	0.60	−2.68
Self-Reported Good Mental Health (Ref.: Bad/fair) ***	−5.93	0.55	−10.79
The PSWS Score ***	0.35	0.05	6.70
F = 34.43; R^2^ = 0.20			

** *p* < 0.01. *** *p* < 0.001. Ref. = reference group. PSWS = Problematic Series Watching Scale.

## Data Availability

The data presented in this study are available on reasonable request from the corresponding author. The data are not publicly available due to ethical requirements.

## Appendix A

**Table A1:** The Problematic Series Watching Scale (PSWS), English version.

1: Never. 2: Rarely. 3: Sometimes. 4: Often. 5: Always.
During the last year, how often have you:
1. thought of how you could free up more time to watch series?
2. spent much more time watching series than initially intended?
3. watched series in order to reduce feelings of guilt, anxiety, helplessness and depression?
4. been told by others to cut down on watching series without listening to them?
5. become restless or troubled if you have been prohibited from watching series?
6. ignored your partner, family members, or friends because of series watching?
